# Triaqua­(3-carb­oxy-5-sulfonatobenzoato-κ*O* ^1^)(1,10-phenanthroline-κ^2^ *N*,*N*′)cobalt(II) monohydrate

**DOI:** 10.1107/S1600536808019843

**Published:** 2008-07-05

**Authors:** Bing-Yu Zhang, Jing-Jing Nie, Duan-Jun Xu

**Affiliations:** a Department of Chemistry, Zhejiang University, Hangzhou 310027, People’s Republic of China

## Abstract

In the title compound, [Co(C_8_H_4_O_7_S)(C_12_H_8_N_2_)(H_2_O)_3_]·H_2_O, the Co^II^ cation is coordinated by one sulfoisophthalate dianion, one bidentate phenathroline (phen) mol­ecule and three water mol­ecules in a distorted cis-CoN_2_O_4_ octa­hedral geometry. In the crystal structure, aromatic π–π stacking occurs [centroid–centroid distances 3.7630 (14) and 3.7269 (15) Å], as well as an extensive O—H⋯O and C—H⋯O hydrogen-bonding network

## Related literature

For related structures, see: Li *et al.* (2005 ▶); Liu *et al.* (2006 ▶).
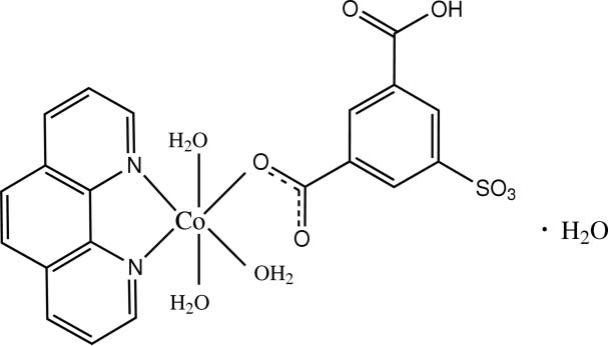


## Experimental

### 

#### Crystal data

[Co(C_8_H_4_O_7_S)(C_12_H_8_N_2_)(H_2_O)_3_]·H_2_O
*M* *_r_* = 555.37
Monoclinic, 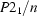

*a* = 10.9968 (13) Å
*b* = 13.9358 (18) Å
*c* = 15.870 (2) Å
β = 109.645 (14)°
*V* = 2290.4 (5) Å^3^
*Z* = 4
Mo *K*α radiation
μ = 0.91 mm^−1^
*T* = 295 (2) K
0.36 × 0.24 × 0.20 mm

#### Data collection

Rigaku R-AXIS RAPID IP diffractometer
Absorption correction: multi-scan (*ABSCOR*; Higashi, 1995 ▶) *T*_min_ = 0.740, *T*_max_ = 0.835
25103 measured reflections
4490 independent reflections
3416 reflections with *I* > 2σ(*I*)
*R*_int_ = 0.051

#### Refinement

*R*[*F* ^2^ > 2σ(*F* ^2^)] = 0.035
*wR*(*F* ^2^) = 0.095
*S* = 1.06
4490 reflections
316 parameters
H-atom parameters constrained
Δρ_max_ = 0.40 e Å^−3^
Δρ_min_ = −0.28 e Å^−3^

### 

Data collection: *PROCESS-AUTO* (Rigaku, 1998 ▶); cell refinement: *PROCESS-AUTO*; data reduction: *CrystalStructure* (Rigaku/MSC, 2002 ▶); program(s) used to solve structure: *SIR92* (Altomare *et al.*, 1993 ▶); program(s) used to refine structure: *SHELXL97* (Sheldrick, 2008 ▶); molecular graphics: *ORTEP-3* (Farrugia, 1997 ▶); software used to prepare material for publication: *WinGX* (Farrugia, 1999 ▶).

## Supplementary Material

Crystal structure: contains datablocks I, global. DOI: 10.1107/S1600536808019843/hb2753sup1.cif

Structure factors: contains datablocks I. DOI: 10.1107/S1600536808019843/hb2753Isup2.hkl

Additional supplementary materials:  crystallographic information; 3D view; checkCIF report

## Figures and Tables

**Table 1:** Selected bond lengths (Å)

Co—O1	2.0730 (16)
Co—O5	2.1070 (17)
Co—O6	2.1663 (17)
Co—O7	2.1277 (16)
Co—N1	2.1198 (19)
Co—N2	2.141 (2)

**Table 2:** Hydrogen-bond geometry (Å, °)

*D*—H⋯*A*	*D*—H	H⋯*A*	*D*⋯*A*	*D*—H⋯*A*
O1*W*—H1*A*⋯O6^i^	0.95	2.11	2.881 (3)	137
O1*W*—H1*B*⋯O11^ii^	0.96	1.93	2.873 (3)	165
O4—H4*A*⋯O1*W*	0.93	1.71	2.621 (3)	166
O5—H5*A*⋯O13^iii^	0.84	1.86	2.695 (3)	175
O5—H5*B*⋯O3^iv^	0.86	1.94	2.798 (2)	174
O6—H6*A*⋯O3^v^	0.81	2.08	2.803 (2)	149
O6—H6*B*⋯O12^vi^	0.85	1.95	2.790 (3)	173
O7—H7*A*⋯O2	0.86	1.73	2.579 (3)	168
O7—H7*B*⋯O11^iii^	0.84	2.03	2.859 (2)	172
C1—H1⋯O5^iv^	0.93	2.56	3.249 (3)	131
C2—H2⋯O13^vii^	0.93	2.59	3.506 (4)	167
C3—H3⋯O2^vii^	0.93	2.48	3.399 (3)	168
C6—H6⋯O1*W*^viii^	0.93	2.59	3.391 (4)	145
C9—H9⋯O12^viii^	0.93	2.47	3.373 (4)	164

Symmetry codes: (i) 
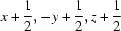
; (ii) 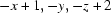; (iii) 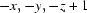; (iv) 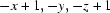; (v) 
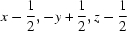
; (vi) 
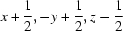
; (vii) 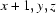; (viii) 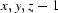.

**Fig. 1.**
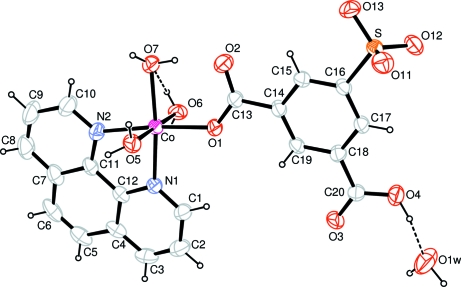
The molecular structure of (I) with 40% probability displacement ellipsoids (arbitrary spheres for H atoms). Dashed lines indicate hydrogen bonding.

**Fig. 2.**
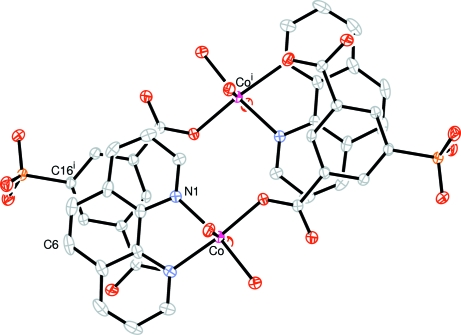
A diagram showing π–π stacking between aromatic rings [symmetry code: (i) 1 - x, -y, 1 - z].

## supplementary crystallographic information

### Comment

As part of our ongoing studies of aromatic π–π stacking in coordination complexes (Li *et al.*,  2005; Liu *et al.*,  2006), the title Co^II^ compound, (I), incorporating the sulfoisophthalate ligand has been prepared and its crystal structure is reported here (Fig. 1).

The Co^II^ cation in (I) is coordinated by one sulfoisophthalate dianion, one bidentate phenathroline (phen) molecule and three water molecules in a distorted CoN_2_O_4_ octahedral geometry (Table 1). Among the two carboxyl groups of the sulfoisophthalate, the C13-carboxyl group is deprotonated and the difference between C13—O1 and C13—O2 bond distances is small whereas the C20-carboxyl group is not deprotonated and the difference between the C20—O3 and C20—O4 bond distances is larger (Table 1).

This is in agreement with those found in related structures, e.g. *catena*-((µ_3_-5-carboxy-3-sulfonatobenzoato)aqua(phenanthroline)lead(II) monohydrate (Li *et al.*, 2005) and bis(µ_2_-aqua)hexaaquabis(5-sulfoisophthalato)dicadmium(II) (Liu *et al.*, 2006). The C13-carboxyl group is hydrogen bonded (as an acceptor) to the coordinated water molecule while the C20-carboxylo group is hydrogen bonded (as a donor) to the uncoordinated water molecule (Fig. 1). An extensive O—H···O and C—H···O hydrogen bonding network helps to consolidate the packing (Table 2).

A partially overlapped arrangement is observed between nearly parallel phen ring system and the benzene ring of the sulfoisophthalate dianion from an adjacent complex (Fig. 2). The centroid-to-centroid distances of 3.7630 (14) Å between the N1-pyridine and C16^i^-benzene rings and 3.7269 (15) Å between the C6-benzene and C16^i^-benzene rings [symmetry code: (i) 1 - *x*, -*y*, 1 - *z*] indicate the existence of π–π stacking between phen and sulfoisophthalate of the adjacent molecule.

### Experimental

A water–ethanol solution (15 ml, 2:1 v/v) containing monosodium 5-sulfoisophthalate (0.27 g, 1 mmol), sodium carbonate (0.053 g, 0.5 mmol), 1,10-phenanthroline (0.10 g, 0.5 mmol) and cobalt nitrate hexahydrate (0.29 g, 1 mmol) was refluxed for 3 h. After cooling to room temperature the solution was filtered. Red prisms of (I) were obtained from the filtrate after one week.

### Refinement

The carboxyl H and water H atoms were located in a difference Fourier map and refined as riding in as-found relative positions with *U*_iso_(H) = 1.5*U*_eq_(O). Aromatic H atoms were placed in calculated positions with C—H = 0.93 Å and refined in riding mode with *U*_iso_(H) = 1.2*U*_eq_(C).

### Crystal data

**Table:**

[Co(C_8_H_4_O_7_S)(C_12_H_8_N_2_)(H_2_O)_3_]·H_2_O	*F*_000_ = 1140
*M**_r_* = 555.37	*D*_x_ = 1.611 Mg m^−^^3^
Monoclinic, *P*2_1_/*n*	Mo *K*α radiation λ = 0.71073 Å
Hall symbol: -P 2yn	Cell parameters from 6846 reflections
*a* = 10.9968 (13) Å	θ = 2.0–25.0º
*b* = 13.9358 (18) Å	µ = 0.91 mm^−^^1^
*c* = 15.870 (2) Å	*T* = 295 (2) K
β = 109.645 (14)º	Prism, red
*V* = 2290.4 (5) Å^3^	0.36 × 0.24 × 0.20 mm
*Z* = 4	

### Data collection

**Table:**

Rigaku R-AXIS RAPID IP diffractometer	4490 independent reflections
Radiation source: fine-focus sealed tube	3416 reflections with *I* > 2σ(*I*)
Monochromator: graphite	*R*_int_ = 0.051
Detector resolution: 10.0 pixels mm^-1^	θ_max_ = 26.0º
*T* = 295(2) K	θ_min_ = 2.0º
ω scans	*h* = −13→13
Absorption correction: multi-scan(ABSCOR; Higashi, 1995)	*k* = −17→16
*T*_min_ = 0.740, *T*_max_ = 0.835	*l* = −18→19
25103 measured reflections	

### Refinement

**Table:**

Refinement on *F*^2^	Secondary atom site location: difference Fourier map
Least-squares matrix: full	Hydrogen site location: inferred from neighbouring sites
*R*[*F*^2^ > 2σ(*F*^2^)] = 0.035	H-atom parameters constrained
*wR*(*F*^2^) = 0.095	*w* = 1/[σ^2^(*F*_o_^2^) + (0.0494*P*)^2^ + 0.1783*P*] where *P* = (*F*_o_^2^ + 2*F*_c_^2^)/3
*S* = 1.06	(Δ/σ)_max_ < 0.001
4490 reflections	Δρ_max_ = 0.40 e Å^−^^3^
316 parameters	Δρ_min_ = −0.28 e Å^−^^3^
Primary atom site location: structure-invariant direct methods	Extinction correction: none

### Special details

**Table:**

Geometry. All e.s.d.'s (except the e.s.d. in the dihedral angle between two l.s. planes) are estimated using the full covariance matrix. The cell e.s.d.'s are taken into account individually in the estimation of e.s.d.'s in distances, angles and torsion angles; correlations between e.s.d.'s in cell parameters are only used when they are defined by crystal symmetry. An approximate (isotropic) treatment of cell e.s.d.'s is used for estimating e.s.d.'s involving l.s. planes.
Refinement. Refinement of *F*^2^ against ALL reflections. The weighted *R*-factor *wR* and goodness of fit *S* are based on *F*^2^, conventional *R*-factors *R* are based on *F*, with *F* set to zero for negative *F*^2^. The threshold expression of *F*^2^ > σ(*F*^2^) is used only for calculating *R*-factors(gt) *etc*. and is not relevant to the choice of reflections for refinement. *R*-factors based on *F*^2^ are statistically about twice as large as those based on *F*, and *R*- factors based on ALL data will be even larger.

### Fractional atomic coordinates and isotropic or equivalent isotropic displacement parameters (Å^2^)

**Table:**

	*x*	*y*	*z*	*U*_iso_*/*U*_eq_	
Co	0.32537 (3)	0.13651 (2)	0.35952 (2)	0.03134 (12)	
S	0.05040 (5)	0.06815 (4)	0.78134 (4)	0.03161 (16)	
N1	0.52786 (18)	0.12769 (13)	0.38882 (13)	0.0321 (4)	
N2	0.3431 (2)	0.14723 (15)	0.22955 (13)	0.0387 (5)	
O1	0.32643 (15)	0.12435 (12)	0.48995 (11)	0.0367 (4)	
O2	0.11757 (17)	0.14036 (17)	0.47225 (13)	0.0674 (7)	
O3	0.63866 (15)	0.09338 (13)	0.80484 (11)	0.0418 (4)	
O4	0.55844 (17)	0.09929 (17)	0.91585 (12)	0.0585 (6)	
H4A	0.6392	0.0966	0.9595	0.088*	
O5	0.30048 (15)	−0.01239 (12)	0.33733 (11)	0.0386 (4)	
H5A	0.2252	−0.0345	0.3241	0.058*	
H5B	0.3245	−0.0352	0.2950	0.058*	
O6	0.35517 (15)	0.28948 (12)	0.38099 (11)	0.0388 (4)	
H6A	0.2923	0.3108	0.3420	0.058*	
H6B	0.4197	0.3111	0.3694	0.058*	
O7	0.12326 (15)	0.16385 (12)	0.31242 (11)	0.0405 (4)	
H7A	0.1099	0.1588	0.3629	0.061*	
H7B	0.0742	0.1278	0.2734	0.061*	
O11	0.05105 (16)	−0.03233 (12)	0.80684 (11)	0.0435 (4)	
O12	0.07483 (16)	0.13193 (13)	0.85730 (12)	0.0444 (5)	
O13	−0.06484 (15)	0.09361 (14)	0.70756 (12)	0.0465 (5)	
O1W	0.7986 (2)	0.1114 (2)	1.02278 (15)	0.0893 (9)	
H1A	0.8596	0.1380	0.9985	0.134*	
H1B	0.8576	0.0794	1.0741	0.134*	
C1	0.6184 (2)	0.11688 (18)	0.46861 (17)	0.0395 (6)	
H1	0.5929	0.1065	0.5181	0.047*	
C2	0.7504 (3)	0.12047 (19)	0.4809 (2)	0.0488 (7)	
H2	0.8108	0.1121	0.5377	0.059*	
C3	0.7901 (2)	0.13620 (18)	0.4096 (2)	0.0482 (7)	
H3	0.8777	0.1396	0.4175	0.058*	
C4	0.6977 (2)	0.14729 (16)	0.32399 (19)	0.0393 (6)	
C5	0.7295 (3)	0.16124 (19)	0.2445 (2)	0.0497 (7)	
H5	0.8156	0.1661	0.2486	0.060*	
C6	0.6364 (3)	0.16742 (19)	0.1640 (2)	0.0527 (8)	
H6	0.6597	0.1761	0.1134	0.063*	
C7	0.5023 (3)	0.16096 (18)	0.15414 (18)	0.0446 (7)	
C8	0.4010 (3)	0.1641 (2)	0.07230 (19)	0.0596 (8)	
H8	0.4191	0.1708	0.0194	0.071*	
C9	0.2766 (3)	0.1574 (2)	0.0696 (2)	0.0637 (9)	
H9	0.2094	0.1578	0.0151	0.076*	
C10	0.2509 (3)	0.1500 (2)	0.14981 (19)	0.0537 (8)	
H10	0.1653	0.1468	0.1473	0.064*	
C11	0.4678 (2)	0.15056 (17)	0.23164 (17)	0.0349 (6)	
C12	0.5665 (2)	0.14201 (16)	0.31698 (16)	0.0323 (5)	
C13	0.2328 (2)	0.12619 (17)	0.51846 (16)	0.0348 (6)	
C14	0.2602 (2)	0.10967 (16)	0.61750 (15)	0.0305 (5)	
C15	0.1576 (2)	0.09694 (17)	0.64978 (16)	0.0320 (5)	
H15	0.0731	0.0971	0.6102	0.038*	
C16	0.1814 (2)	0.08409 (16)	0.74032 (15)	0.0293 (5)	
C17	0.3073 (2)	0.08407 (16)	0.80040 (16)	0.0317 (5)	
H17	0.3226	0.0759	0.8613	0.038*	
C18	0.4098 (2)	0.09629 (17)	0.76915 (15)	0.0312 (5)	
C19	0.3862 (2)	0.10878 (17)	0.67763 (16)	0.0313 (5)	
H19	0.4551	0.1165	0.6567	0.038*	
C20	0.5461 (2)	0.09713 (18)	0.83179 (16)	0.0351 (6)	

### Atomic displacement parameters (Å^2^)

**Table:**

	*U*^11^	*U*^22^	*U*^33^	*U*^12^	*U*^13^	*U*^23^
Co	0.02507 (18)	0.0439 (2)	0.0262 (2)	−0.00116 (13)	0.01016 (14)	0.00125 (14)
S	0.0246 (3)	0.0425 (3)	0.0300 (3)	−0.0027 (2)	0.0122 (3)	−0.0003 (3)
N1	0.0287 (10)	0.0399 (11)	0.0287 (11)	0.0004 (8)	0.0110 (9)	0.0012 (9)
N2	0.0379 (12)	0.0501 (13)	0.0271 (12)	0.0012 (10)	0.0095 (10)	0.0028 (9)
O1	0.0289 (9)	0.0581 (11)	0.0255 (9)	0.0008 (7)	0.0122 (7)	0.0025 (8)
O2	0.0307 (10)	0.141 (2)	0.0324 (11)	0.0098 (11)	0.0125 (9)	0.0141 (12)
O3	0.0264 (9)	0.0628 (11)	0.0378 (10)	−0.0036 (8)	0.0130 (8)	−0.0111 (9)
O4	0.0326 (10)	0.1136 (17)	0.0280 (10)	−0.0016 (11)	0.0086 (8)	−0.0058 (11)
O5	0.0324 (9)	0.0489 (10)	0.0370 (10)	−0.0058 (7)	0.0149 (8)	−0.0054 (8)
O6	0.0271 (8)	0.0478 (10)	0.0438 (10)	0.0009 (7)	0.0149 (8)	0.0058 (8)
O7	0.0306 (9)	0.0577 (11)	0.0318 (10)	−0.0018 (8)	0.0085 (8)	−0.0016 (8)
O11	0.0412 (10)	0.0457 (10)	0.0476 (11)	−0.0046 (8)	0.0205 (9)	0.0054 (9)
O12	0.0374 (10)	0.0588 (12)	0.0421 (11)	−0.0031 (8)	0.0200 (9)	−0.0150 (9)
O13	0.0248 (9)	0.0721 (13)	0.0401 (11)	−0.0009 (8)	0.0077 (8)	0.0100 (9)
O1W	0.0410 (12)	0.174 (3)	0.0463 (14)	−0.0109 (14)	0.0059 (10)	0.0314 (15)
C1	0.0319 (13)	0.0495 (15)	0.0355 (15)	0.0006 (11)	0.0093 (12)	0.0020 (12)
C2	0.0343 (14)	0.0544 (17)	0.0490 (18)	0.0024 (12)	0.0025 (13)	0.0004 (14)
C3	0.0264 (13)	0.0477 (16)	0.071 (2)	0.0014 (11)	0.0164 (14)	−0.0012 (14)
C4	0.0380 (14)	0.0310 (13)	0.0588 (18)	0.0015 (10)	0.0291 (14)	0.0002 (12)
C5	0.0503 (17)	0.0424 (16)	0.072 (2)	0.0004 (13)	0.0416 (17)	−0.0008 (14)
C6	0.077 (2)	0.0411 (15)	0.064 (2)	0.0034 (14)	0.0561 (19)	0.0034 (14)
C7	0.0640 (19)	0.0398 (15)	0.0406 (16)	0.0052 (13)	0.0316 (15)	0.0040 (12)
C8	0.089 (3)	0.063 (2)	0.0345 (17)	0.0117 (18)	0.0306 (17)	0.0098 (14)
C9	0.078 (2)	0.078 (2)	0.0265 (16)	0.0079 (18)	0.0057 (16)	0.0064 (15)
C10	0.0482 (17)	0.072 (2)	0.0343 (16)	0.0018 (15)	0.0059 (14)	0.0050 (14)
C11	0.0438 (15)	0.0335 (13)	0.0340 (14)	0.0020 (10)	0.0217 (12)	0.0021 (10)
C12	0.0350 (13)	0.0287 (12)	0.0389 (15)	−0.0009 (10)	0.0199 (12)	−0.0017 (10)
C13	0.0285 (13)	0.0493 (15)	0.0285 (13)	−0.0020 (11)	0.0123 (11)	0.0011 (11)
C14	0.0306 (12)	0.0347 (12)	0.0286 (13)	−0.0025 (10)	0.0132 (11)	−0.0027 (10)
C15	0.0261 (12)	0.0389 (13)	0.0317 (13)	−0.0010 (10)	0.0108 (10)	−0.0026 (11)
C16	0.0277 (12)	0.0329 (12)	0.0305 (13)	−0.0019 (9)	0.0139 (10)	−0.0022 (10)
C17	0.0305 (12)	0.0397 (14)	0.0276 (13)	−0.0024 (10)	0.0134 (11)	−0.0007 (10)
C18	0.0287 (12)	0.0345 (12)	0.0298 (13)	−0.0038 (10)	0.0089 (10)	−0.0022 (10)
C19	0.0270 (12)	0.0401 (13)	0.0320 (13)	−0.0019 (10)	0.0166 (10)	−0.0016 (11)
C20	0.0269 (12)	0.0452 (14)	0.0328 (14)	−0.0028 (11)	0.0096 (11)	−0.0012 (11)

### Geometric parameters (Å, °)

**Table:**

Co—O1	2.0730 (16)	C2—C3	1.361 (4)
Co—O5	2.1070 (17)	C2—H2	0.9300
Co—O6	2.1663 (17)	C3—C4	1.405 (4)
Co—O7	2.1277 (16)	C3—H3	0.9300
Co—N1	2.1198 (19)	C4—C12	1.411 (3)
Co—N2	2.141 (2)	C4—C5	1.431 (4)
S—O11	1.4569 (18)	C5—C6	1.345 (4)
S—O12	1.4482 (18)	C5—H5	0.9300
S—O13	1.4509 (17)	C6—C7	1.432 (4)
S—C16	1.783 (2)	C6—H6	0.9300
N1—C1	1.330 (3)	C7—C8	1.399 (4)
N1—C12	1.359 (3)	C7—C11	1.411 (3)
N2—C10	1.330 (3)	C8—C9	1.357 (4)
N2—C11	1.361 (3)	C8—H8	0.9300
O1—C13	1.257 (3)	C9—C10	1.398 (4)
O2—C13	1.248 (3)	C9—H9	0.9300
O3—C20	1.231 (3)	C10—H10	0.9300
O4—C20	1.295 (3)	C11—C12	1.428 (3)
O4—H4A	0.9257	C13—C14	1.515 (3)
O5—H5A	0.8416	C14—C19	1.393 (3)
O5—H5B	0.8608	C14—C15	1.399 (3)
O6—H6A	0.8129	C15—C16	1.383 (3)
O6—H6B	0.8463	C15—H15	0.9300
O7—H7A	0.8645	C16—C17	1.392 (3)
O7—H7B	0.8385	C17—C18	1.386 (3)
O1W—H1A	0.9530	C17—H17	0.9300
O1W—H1B	0.9630	C18—C19	1.398 (3)
C1—C2	1.399 (4)	C18—C20	1.495 (3)
C1—H1	0.9300	C19—H19	0.9300
			
O1—Co—O5	92.48 (6)	C12—C4—C5	118.9 (3)
O1—Co—N1	97.09 (7)	C6—C5—C4	120.8 (3)
O5—Co—N1	92.72 (7)	C6—C5—H5	119.6
O1—Co—O7	91.14 (6)	C4—C5—H5	119.6
O5—Co—O7	93.21 (6)	C5—C6—C7	121.8 (3)
N1—Co—O7	169.64 (7)	C5—C6—H6	119.1
O1—Co—N2	174.74 (7)	C7—C6—H6	119.1
O5—Co—N2	87.51 (7)	C8—C7—C11	116.7 (3)
N1—Co—N2	77.66 (8)	C8—C7—C6	124.7 (3)
O7—Co—N2	94.11 (7)	C11—C7—C6	118.7 (3)
O1—Co—O6	88.49 (6)	C9—C8—C7	120.5 (3)
O5—Co—O6	178.49 (6)	C9—C8—H8	119.8
N1—Co—O6	86.00 (6)	C7—C8—H8	119.8
O7—Co—O6	87.93 (6)	C8—C9—C10	119.1 (3)
N2—Co—O6	91.43 (7)	C8—C9—H9	120.4
O12—S—O13	112.88 (11)	C10—C9—H9	120.4
O12—S—O11	112.12 (11)	N2—C10—C9	123.1 (3)
O13—S—O11	112.37 (11)	N2—C10—H10	118.5
O12—S—C16	106.31 (11)	C9—C10—H10	118.5
O13—S—C16	105.58 (11)	N2—C11—C7	123.1 (2)
O11—S—C16	106.98 (10)	N2—C11—C12	117.3 (2)
C1—N1—C12	118.0 (2)	C7—C11—C12	119.6 (2)
C1—N1—Co	127.59 (17)	N1—C12—C4	122.8 (2)
C12—N1—Co	114.17 (15)	N1—C12—C11	117.1 (2)
C10—N2—C11	117.5 (2)	C4—C12—C11	120.1 (2)
C10—N2—Co	129.16 (19)	O2—C13—O1	125.8 (2)
C11—N2—Co	113.33 (16)	O2—C13—C14	116.3 (2)
C13—O1—Co	128.88 (16)	O1—C13—C14	118.0 (2)
C20—O4—H4A	120.8	C19—C14—C15	119.1 (2)
Co—O5—H5A	117.7	C19—C14—C13	121.1 (2)
Co—O5—H5B	116.0	C15—C14—C13	119.7 (2)
H5A—O5—H5B	101.8	C16—C15—C14	120.2 (2)
Co—O6—H6A	101.2	C16—C15—H15	119.9
Co—O6—H6B	114.2	C14—C15—H15	119.9
H6A—O6—H6B	105.4	C15—C16—C17	120.6 (2)
Co—O7—H7A	98.2	C15—C16—S	120.11 (17)
Co—O7—H7B	119.3	C17—C16—S	119.32 (17)
H7A—O7—H7B	111.6	C18—C17—C16	119.7 (2)
H1A—O1W—H1B	99.1	C18—C17—H17	120.1
N1—C1—C2	122.5 (2)	C16—C17—H17	120.1
N1—C1—H1	118.7	C17—C18—C19	119.9 (2)
C2—C1—H1	118.7	C17—C18—C20	121.2 (2)
C3—C2—C1	119.8 (3)	C19—C18—C20	119.0 (2)
C3—C2—H2	120.1	C14—C19—C18	120.5 (2)
C1—C2—H2	120.1	C14—C19—H19	119.8
C2—C3—C4	119.5 (2)	C18—C19—H19	119.8
C2—C3—H3	120.3	O3—C20—O4	123.1 (2)
C4—C3—H3	120.3	O3—C20—C18	122.0 (2)
C3—C4—C12	117.3 (2)	O4—C20—C18	114.8 (2)
C3—C4—C5	123.8 (2)		

### Hydrogen-bond geometry (Å, °)

**Table:**

*D*—H···*A*	*D*—H	H···*A*	*D*···*A*	*D*—H···*A*
O1W—H1A···O6^i^	0.95	2.11	2.881 (3)	137
O1W—H1B···O11^ii^	0.96	1.93	2.873 (3)	165
O4—H4A···O1W	0.93	1.71	2.621 (3)	166
O5—H5A···O13^iii^	0.84	1.86	2.695 (3)	175
O5—H5B···O3^iv^	0.86	1.94	2.798 (2)	174
O6—H6A···O3^v^	0.81	2.08	2.803 (2)	149
O6—H6B···O12^vi^	0.85	1.95	2.790 (3)	173
O7—H7A···O2	0.86	1.73	2.579 (3)	168
O7—H7B···O11^iii^	0.84	2.03	2.859 (2)	172
C1—H1···O5^iv^	0.93	2.56	3.249 (3)	131
C2—H2···O13^vii^	0.93	2.59	3.506 (4)	167
C3—H3···O2^vii^	0.93	2.48	3.399 (3)	168
C6—H6···O1W^viii^	0.93	2.59	3.391 (4)	145
C9—H9···O12^viii^	0.93	2.47	3.373 (4)	164

Symmetry codes: (i) *x*+1/2, −*y*+1/2, *z*+1/2; (ii) −*x*+1, −*y*, −*z*+2; (iii) −*x*, −*y*, −*z*+1; (iv) −*x*+1, −*y*, −*z*+1; (v) *x*−1/2, −*y*+1/2, *z*−1/2; (vi) *x*+1/2, −*y*+1/2, *z*−1/2; (vii) *x*+1, *y*, *z*; (viii) *x*, *y*, *z*−1.
